# Secondhand smoke exposure during pregnancy: Nicotine metabolites in maternal urine and breast milk as biomarkers of infant birth weight and length, a cross-sectional study

**DOI:** 10.18332/tid/226408

**Published:** 2026-09-17

**Authors:** Jadsada Kunno, Panu Pimviriyakul, Titaporn Luangwilai, Parichat Ong-artborirak, Saowanee Sematong, Thanapong Chaichana, Budsaba Wiriyasirivaj, Mark Gregory Robson

**Affiliations:** 1 Department of Research and Medical Innovation, Faculty of Medicine Vajira Hospital, Navamindradhiraj University, Bangkok, Thailand; 2 Department of Biochemistry, Faculty of Science, Kasetsart University, Bangkok, Thailand; 3 College of Public Health Sciences, Chulalongkorn University, Bangkok, Thailand; 4 Department of Obstetrics and Gynecology, Faculty of Medicine Vajira Hospital, Navamindradhiraj University, Bangkok, Thailand; 5 School of Environmental and Biological Sciences, Rutgers University, New Jersey, United States

**Keywords:** secondhand smoke, pregnancy, nicotine metabolites, breast milk, infant birth

## Abstract

**INTRODUCTION:**

Exposure to secondhand smoke (SHS) during pregnancy (P-SHS) has adverse effects on breast milk composition and infant birth outcomes. However, evidence clarifying the exact relationship between nicotine exposure and specific infant outcomes is currently limited. This study aims to explore the association between nicotine metabolite levels – in the urine and breast milk of mothers exposed to SHS – and infant birth weight and length, as well as to assess how P-SHS-related variables modify exposure risk.

**METHODS:**

This study was a cross-sectional study of first pregnancies from April to December 2025 in an urban low-income community in Bangkok, Thailand. This study used multi-stage purposive sampling to recruit pregnant women and their infants who were highly exposed to secondhand smoke. Nicotine metabolites were measured in mothers’ third-trimester urine and post-delivery breast milk samples, and infants’ birth weight and length were measured at the participants’ households. All measurements were done after participants provided written consent and had answered the questionnaire. Multiple linear regression was used to assess the association between maternal nicotine metabolites and infant birth outcomes and between P-SHS exposure and participant nicotine levels.

**RESULTS:**

This study involved 95 pregnant participants exposed to household smoking and their 95 infants. Prenatal maternal urine nicotine metabolite level was significantly associated with breast milk nicotine concentration (p<0.001). Infant weight (p=0.048) and length (p=0.034) were significantly associated with prenatal maternal urine nicotine metabolite levels. Several P-SHS variables reflecting greater SHS exposure, such as smoking in the living room and bedroom (p<0.01), were significantly associated with higher nicotine metabolite levels in prenatal maternal urine.

**CONCLUSIONS:**

Prenatal P-SHS exposure elevated nicotine metabolite concentrations in maternal urine and breast milk, which were significantly associated with infant birth weight and length.

## INTRODUCTION

Exposure to secondhand smoke (SHS) during pregnancy is directly harmful to fetuses, contributing to increased risks for serious complications, such as premature birth, miscarriage, low birth weight, and birth defects^1,2^. These complications arise because nicotine, which readily diffuses into fetal blood, amniotic fluid, and breast milk, negatively affects neurological development. The fetuses and infants of mothers who smoke are consequently at elevated risk for poor health^2^. Prenatal SHS (P-SHS) exposure is also linked to adverse birth outcomes, including low birth weight^3,4^, shorter birth length^5,6^, and preterm birth. Robust public health interventions are therefore needed to ensure that women are protected against P-SHS exposure^7^. In addition, although 6 months of feeding infants exclusively with breast milk is widely known to reduce adverse events in infants, public health efforts are hampered by the limited evidence of how SHS exposure specifically affects breastfeeding initiation, prevalence, and duration^2^. However, breast milk concentrations of nicotine and cotinine are related directly to maternal smoking habits, particularly the number of cigarettes consumed per day and the interval between smoking and breastfeeding^8^. Mothers should be educated about the secretion of harmful cigarette chemicals into breast milk and are strongly encouraged to avoid smoking while lactating^9^. Although breastfeeding benefits generally outweigh the risk of nicotine exposure, mothers should abstain completely from all smoke exposure during pregnancy^9^.

Shifts of populations from rural to urban areas in Thailand have accelerated urban growth, which has led to overcrowding in low-income areas; high rates of smoking in such areas have been reported^10^. Data from a previous study in a low-income urban community in Bangkok, Thailand, indicated that tobacco exposure is the dominant environmental factor in neonatal morbidity. This community in Bangkok was selected for the earlier study because it had a large population of active smokers, daily consumption was heavy (averaging over 10 cigarettes daily), and indoor household smoking was prevalent (82.8%)^11^. Furthermore, the high density of households in this area exacerbated the exposure risk, inasmuch as poor ventilation can directly increase exposure to indoor air pollutants^12^.

According to the National Institutes of Health in the United States, the reported probability of daily SHS exposure is higher for pregnant women in urban households than for their rural counterparts^13^. This evidence influenced our interest in P-SHS exposure within a low-income urban community. The existing evidence regarding the association between nicotine exposure and infant outcomes, however, is limited. Nicotine and cotinine concentrations in breast milk directly reflect maternal smoking habits (daily consumption and feeding interval)^8^.

The primary research questions concerned the extent to which nicotine metabolite levels in prenatal urine and breast milk are associated with infant birth weight and length and how P-SHS-related variables are involved in modifying this exposure risk (Supplemental file Material 1). Consequently, this study hypothesized that higher nicotine metabolite levels in maternal urine or breast milk, or both, are associated with infant birth weight and length. Moreover, variables indicative of greater SHS exposure (e.g. higher frequency of family smoking and longer cohabitation with smokers) are associated with significant increases in nicotine metabolite levels during pregnancy. This study’s specific aims were: 1) to explore nicotine metabolite levels in mothers’ urine and breast milk; 2) to determine how P-SHS-related variables (such as living with heavy smokers and family smoking frequency) affect pregnancy nicotine metabolite levels (exposure risk) in this population. In addition, the second aim is to determine whether nicotine metabolite levels in mothers’ urine and breast milk are associated with infant birth weight and length. In addition, maternal nicotine exposure restricts vital nutrients and disrupts fetal cellular growth; it is a potent teratogen that crosses the placenta, and it ultimately reduces infant birth weight and length^14,15^.

## METHODS

### Study design

This cross-sectional study was conducted as a subproject of the larger initiative, ‘The Effect on Smoking and the Development of Mobile Health Applications to Promote Smoking-Free in Urban Communities, Bangkok, Thailand’, which was supported by Thailand Science Research and Innovation (TSRI; Grant No. FRB670080/0468).

Ethics approval for this study was granted by the Institutional Review Board of the Faculty of Medicine at Vajira Hospital, Navamindradhiraj University, Bangkok, Thailand (COA 016/2566). In all procedures, we adhered strictly to the Declaration of Helsinki and to relevant guidelines and regulations, such as the guidelines of the Belmont Report, the Council for International Organizations of Medical Sciences, and the International Council of Harmonization-Good Clinical Practice (ICH-GCP).

For this study, pregnant participants were recruited between April and December 2025 from the Bangkok low-income urban community previously described^11^. This study used multi-stage purposive sampling to recruit pregnant women exposed to secondhand smoke. First, a community survey identified pregnant women and assessed their household smoking dynamics. Second, we selected those living in high-risk households, characterized by heavy household smoking during an active pregnancy. All mothers provided written informed consent for themselves and their infants to participate, and all mothers completed relevant questionnaires.

### Data collection

P-SHS-related information, specifically focusing on household SHS exposure, was systematically collected in four phases (Supplemental file Material 2). The first phase focused on identifying the target population and collecting detailed self-reported data on household smoking. The following steps were performed: 1) a community survey, conducted by community leaders, to determine the overall number of pregnancies in this community; 2) identification and documentation of eligible pregnant participants whose household included a person who smoked (e.g. husband, in-law, or other relative); 3) determination of the frequency of smoking by household members (e.g. number of days [typically, 5–7 days] per week); 4) quantification of the average number of cigarettes smoked daily by household members, especially those who smoked heavily; 5) recording of the duration of cohabitation (years) between the pregnant participants and household members who smoked; and 6) determination of the smoking locations within the household (e.g. indoors/outdoors; specific rooms, such as the bedroom, living room, or kitchen).

In the second phase, the documented information from the participants was collected at three sequential stages: during pregnancy (interview data on SHS exposure and urine samples during the third trimester [27–40 weeks]), after childbirth (breast milk samples approximately 1 week after childbirth), and during the neonatal period (weighing and measuring of infants). The systematic collection plan for these data points was thoroughly explained to all participants before they provided written informed consent. In the third phase, we documented the initial exposure biomarkers and self-reported risk factors, using questionnaire data (comprising the detailed SHS exposure information gathered in the first phase) and third trimester urine samples for nicotine metabolite analysis. In the fourth phase, breast milk samples were collected, and infant outcomes (birth weight and length) were documented from the prenatal record book and medical records.

### Participant recruitment

The study participants were classified according to stage and data contribution during the second phase (Supplemental file Material 2). Recruitment was based on strict inclusion criteria: non-smoking women aged >18 years, in the third trimester of their first pregnancy (27–40 weeks), living in the low-income community with someone who smoked (to determine SHS exposure), and with a documented history of good health (confirmed via medical records). This study collected data from questionnaires and prenatal urine samples. All data collection occurred at the participants’ respective household locations. To determine the required sample size, we used G*Power software (the effect size is 0.34) to estimate the population of children residing with parents classified as smoking cigarettes heavily in household areas^11,12^.

### Questionnaire

The questionnaire used in this study (estimated completion time: 15–20 minutes) was developed and validated by an expert research team (content validity index: 0.80–1.00). The questionnaire covered three main areas: 1) pregnancy-related demographics (age, education level, income, time of conception, prenatal and perinatal complications, trimester of pregnancy); 2) P-SHS exposure (self-reported risk factors, including family smoking frequency/intensity, smoker status, length of cohabitation with someone who smoked, location and type [e.g. cigarettes] of smoking); and 3) infant outcomes (gender, weight, and length, provided by the mothers).

### Urine and breast milk collection

Participants were provided with a labeled polyethylene bottle and detailed instructions for urine collection. Approximately 50 mL of morning voiding urine was collected. To ensure sample integrity, samples were immediately transferred from the bottles into screw-cap polyethylene tubes, sealed within a zip-lock plastic bag, and kept in an icebox during transportation to the laboratory. Approximately 1 week after childbirth, participants collected 1 mL of breast milk, which was placed into a labeled opaque amber glass bottle (to protect light-sensitive analytes). Immediately after collection, the bottle was sealed and stored in an icebox during transport to the laboratory. To preserve stability, both urine and breast milk samples were stored at 20°C in a freezer on dry ice immediately upon arrival at the laboratory. All samples were then shipped for analysis within 2 days.

### Analysis of nicotine metabolites in urine and breast milk

To analyze nicotine metabolites in the thawed urine and breast milk samples, we used an enzyme-linked immunosorbent assay kit (catalog number KA0930, v.08; Abnova USA, Atlanta, GA, USA). This study adhered strictly to the manufacturer’s instructions, and to ensure reliability, the process was repeated three times, in accordance with methods established in prior studies^11,12^. The nicotine standard curves were linear from 0 to 100 ng/mL. Recovery tests demonstrated 99% accuracy in urine samples at the standard concentrations specified by the enzyme-linked immunosorbent assay (ELISA) kit. The method detection limits were 0.01–0.1 ng/mL of urine and breast milk samples.

### Infant birth weight and length

Infant birth weight and length were documented from the prenatal record book and medical records and collected concurrently with the breast milk sample.

### Potential covariates

Maternal BMI and infant gender. A pregnancy weight gain calculator, based on Institute of Medicine guidelines^16^, was used to estimate healthy weight gain according to maternal BMI. Inputs included height, pre-pregnancy weight, current weight, and gestational age (weeks 27–40).

### Statistical analysis

Summary statistics, including means and standard deviations, were calculated to describe the characteristics of the participants. Multiple linear regression models were used to analyze the association between the levels of maternal nicotine metabolites (in urine and breast milk) and infant birth weight and length, with adjustment for maternal BMI and infant gender, and multiple linear regression to analyze the association between P-SHS-related variables (e.g. family smoking intensity/frequency) and the nicotine levels among the SHS-exposed participants, with adjustment for age and BMI maternal. To perform statistical analysis, we used SPSS Statistics, Version 30 (IBM Corp., Armonk, NY, USA); a p<0.05 denoted significance.

## RESULTS

### Characteristics of P-SHS exposure

The data from 95 pregnant and 95 infant participants included information about maternal living conditions, infant measurements, SHS exposure, and nicotine metabolite concentrations in urine and breast milk (Table 1). The average age of the participants was 32.50 years. The majority were vendors (45.3%), had completed high school (63.2%), and reported incomes of >15000 Thai Baht (61.1%). The participants generally reported uncomplicated pregnancies: the majority were naturally conceived (88.4%), had no history of miscarriage (85.3%), and experienced no complications during pregnancy or delivery (85.3%). Furthermore, the majority (55.8%) anticipated delivery at full-term (39–40 weeks), and their average weights were 55.66 kg before pregnancy and 65.94 kg during the third trimester (27–40 weeks).

**Table 1:** Characteristics of P-SHS participants (N=95) and infants (N=95)

*Characteristics*	*n (%)*
**Sociodemographic information**	
Age (years), mean ± SD	32.50 ± 4.35
**Occupation**	
Unemployed	18 (18.9)
General employee	19 (20.0)
Vendor	43 (45.3)
Permanent employee	15 (15.8)
**Education level**	
<Hight school	9 (9.5)
Hight school	60 (63.2)
≥Bachelor’s degree	26 (27.3)
**Monthly income** (THB)	
<15000	37 (38.9)
≥15000	58 (61.1)
**Pregnancy information**	
**Type of conception**	
Naturally conceived	84 (88.4)
Not naturally conceived	11 (11.6)
**Number of miscarriages**	
0	81 (85.3)
1	14 (14.7)
**Current complications**	
No	81 (85.3)
Yes	14 (14.7)
**Previous complications**	
No	81 (85.3)
Yes	14 (14.7)
**Pregnancy stage**, mean ± SD	
Weight before pregnancy (kg)	55.66 ± 2.32
Height (cm)	163.28 ± 4.55
BMI (kg/m^2^)	20.88 ± 0.76
**Third trimester** (28–40 weeks), mean ± SD	
Third trimester (weeks)	34.25 ± 1.22
Weight (kg)	65.94 ± 2.68
**Expected date of delivery** (weeks)	
Early-term (37–38)	-
Full-term (39–40)	53 (55.8)
Post-term (>40)	42 (44.2)
**P-SHS information**	
**How many days a week does your family smoke?**	
1	19 (20.0)
2	14 (14.7)
3–4	19 (20.0)
5–7	43 (45.3)
**Family members’ cigarettes/day**	
2–5	13 (13.7)
6–10	21 (22.1)
>11	61 (64.2)
**Family members’ smoker status**	
Husband only	50 (52.6)
Husband and others in family	45 (47.4)
**How long have you lived with a smoker?** (years)	
2	14 (14.7)
3	34 (35.8)
4	20 (21.1)
5	27 (28.4)
**Duration of smoking** (years)	
3–4	31 (32.6)
5–7	39 (41.1)
≥8	25 (26.3)
**Areas for smoking in household**	
Bedroom	19 (20.0)
Living room	43 (45.3)
Both	33 (34.7)
**Type of smoking**	
Self-rolled cigarettes	24 (25.3)
Ready-rolled cigarettes	47 (49.4)
Both	24 (25.3)
**Infant information**	
**Gender**	
Male	27 (28.4)
Female	68 (71.6)
**Weight** (g)	
Mean ± SD	2970 ± 137.26
Range	2800–3300
**Length (cm)**	
Mean ± SD	49.15 ± 0.74
Range	48.00–51.00
**Nicotine metabolites concentration** (ng/mL)	
**Urine during the third trimester**	
Mean ± SD	0.70 ± 1.00
Range	0.11–8.52
**Breast milk approximately 1 week after delivery**	
Mean ± SD	0.22 ± 0.46
Range	3.21–0.22

THB: 1000 Thai Baht about US$30.

Most participants experienced regular P-SHS exposure; family members of 45.3% of the participants typically smoked 5–7 days a week, and for >64.2% of the participants family members consumed more than 11 cigarettes daily. The husbands of 52.6% of the participants were the primary smokers, and the husbands of 41.1% had been smoking for 5–7 years. Of the husbands who smoked, 49.4% used self-rolled cigarettes, 34.7% smoked in both the bedroom and living room, and 35.8% had lived with the participants for an average of 3 years. Of the 95 infants, the majority (71.6%) were female. Their anthropometric measures averaged 2970 g for weight and 49.15 cm for length. The average nicotine metabolite concentrations were 0.70 ± 1.00 ng/mL in urine during the third trimester, and 0.22 ± 0.46 ng/mL in breast milk approximately 1 week after delivery.

### Nicotine metabolite levels in maternal urine and breast milk in association with infant birth weight and length

The multiple linear regression analysis focused on nicotine metabolite concentrations in prenatal urine and postnatal breast milk and their associations with infant birth weight and infant length, with adjustment for maternal BMI and infant gender (Table 2). The urine nicotine metabolite levels were significantly and positively associated with breast milk nicotine concentration (β=2.07; 95% CI: 1.93–2.21; p<0.001): specifically, every 1-unit increase in the mother’s urinary metabolite concentration was predictive of a 2.07-unit increase in the concentration found in breast milk. Infant length was significantly but negatively associated with nicotine metabolite levels in prenatal urine (β= -0.18; 95% CI: -0.36 – -0.01; p=0.034): specifically, infant length was reduced by 0.181 units for every 1-unit increase in prenatal nicotine metabolite concentrations. Infant weight was significantly and positively associated with nicotine metabolite levels in maternal urine (β=0.00; p=0.048). However, the regression coefficient (β) of 0.00 simply indicates that the unit of measurement for the metabolites (e.g. 1 ng/mL) is so small that the per-unit change rounds to zero.

**Table 2:** Association between pregnancy urine nicotine metabolite levels and breast milk and infant growth outcomes

*Variables*	*Mean ± SD*	*β (95% CI)*	*p*
Breast milk (ng/mL)	0.22 ± 0.46	2.07 (1.93–2.21)	**<0.001**
Infant weight (g)	2970 ± 137.26	0.00 (0.000–0.00)	**0.048**
Infant length (cm)	49.15 ± 0.74	-0.18 (-0.36 – -0.01)	**0.034**

Data were analyzed using multiple linear regression models were included in the initial multivariable analysis, along with key covariates (maternal BMI and infant gender). Statistically significant level p<0.05.

### Influence of P-SHS-related variables on nicotine metabolite levels

The multiple linear regression analysis focused on the association of P-SHS exposure with nicotine levels (Table 3). Several variables reflecting greater SHS exposure were significantly associated with higher nicotine metabolite levels in prenatal urine: living with family members who smoked 5–7 days a week (β=1.08; 95% CI: 1.28–6.83; p<0.05); living with people who smoked >11 cigarettes daily (β=1.35; 95% CI: 1.62, 9.31; p<0.01); longer cohabitation (≥3 years) with people who smoked (β=1.35; 95% CI: 1.39–10.80; p<0.01); and exposure to SHS in the living room (β 1.546; 95% CI: 1.686–13.046; p<0.01) or in both the bedroom and living room (β=1.51; 95% CI: 1.62–12.61; p<0.01). The analysis revealed that the same P-SHS-related variables were also significantly and positively associated with higher nicotine metabolite levels in breast milk: living with family members who smoked 5–7 days a week (β=6.57; 95% CI: 13.35–38654.02; p<0.01), living with people who smoked >11 cigarettes daily (β=2.40; 95% CI: 1.44–84.35; p<0.05), longer cohabitation (≥3 years) with people who smoked (β=3.67; 95% CI: 1.71–914.47; p<0.05), and exposure to SHS in the living room (β=5.70; 95% CI: 4.65–19392.11); p<0.01) or in both the bedroom and living room (β=6.06; 95% CI: 6.71–27661.57; p<0.01).

**Table 3:** Association between P-SHS variables and nicotine metabolite levels

*P-SHS variables*	*Nicotine metabolite*	
	*Urine* *(ng/mL)*	*Breast milk* *(ng/mL)*
	*β (95% CI)*	*β (95% CI)*
**How many days a week does your family smoke?**		
1 (ref.)	0	0
2	-0.01 (0.35–2.78)	0.27 (0.01–214.91)
3–4	0.01 (0.36–2.84)	0.08 (0.01–191.51)
5–7	1.08 (1.28–6.83)*	6.57 (13.35–38654.02)**
**Family members’ cigarettes/day**		
2–5 (ref.)	0	0
6–10	0.51 (0.63–4.42)	-0.12 (0.07–11.60)
>11	1.35 (1.62–9.31)**	2.40 (1.440–84.35)*
**Family members’ smoker status**		
Husband only (ref.)	0	0
Husband and others in family	0.06 (0.76–1.49)	0.24 (0.55–2.96)
**How long have you lived with a smoker?** (years)		
2 (ref.)	0	0
3	1.35 (1.39–10.80)**	3.67 (1.71–914.47)*
4	0.75 (0.70–6.41)	2.46 (0.43–316.39)
5	0.98 (0.93–7.66)	2.71 (0.59–383.03)
**Duration of smoking** (years)		
3–4 (ref.)	0	0
5–7	0.07 (0.74–1.55)	0.14 (0.50–2.68)
≥8	-0.15 (0.51–1.41)	-0.65 (0.11–2.42)
**Areas for smoking in household**		
Bedroom (ref.)	0	0
Living room	1.54 (1.68–13.04)**	5.70 (4.65–19392.12)**
Both	1.51 (1.62–12.61)**	6.06 (6.71–27661.57)**
**Type of smoking**		
Self-rolled cigarettes (ref.)	0	0
Ready-rolled cigarettes	0.38 (0.81–2.64)	1.07 (0.48–17.78)
Both	0.34 (0.78–2.54)	1.24 (0.57–20.79)

Variables in the univariate analysis, along with key covariates (age and BMI maternal). Data were analyzed using linear regression models. Statistically significant level:

* p<0.05,

** <0.01,

***p<0.001.

## DISCUSSION

This study focused on non-smoking women during the third trimester of their first pregnancy and after childbirth who lived with people who smoked. We recruited these participants from a low-income urban community in Bangkok, Thailand, because of previous findings^11^ of heavy tobacco use in that area (e.g. 67.1% of household smokers consumed ≥10 cigarettes/day and 90.6% used self-rolled cigarettes).

### Nicotine metabolite concentrations in the urine and breast milk of women exposed to SHS

According to an earlier analysis^17^, moderate SHS exposure was confirmed by a nicotine level of at least 0.25 ng/mL; lower levels characterized intermittent or thirdhand smoke exposure. Our study revealed that the average concentration of urinary nicotine metabolites was 0.70 ± 1.00 ng/mL during the third trimester of pregnancy; this level was consistent with the cutoff of 0.25 ng/mL. Pregnant women exposed to SHS exhibited significantly higher urinary nicotine levels than did non-exposed women; this finding was supported by a previous study in which the urinary nicotine concentration was 0.18 ng per mg of creatinine in the SHS-exposed pregnant women and 0.03 ng per mg of creatinine in the non-exposed women^18^. In contrast, another previous study^19^ showed that participants 32 weeks pregnant who had smoked >5 cigarettes in the previous 7 days had a mean nicotine metabolite concentration of 444.5 ± 760.6 ng/mL. Furthermore, pregnant participants engaging in *ad libitum* smoking exhibited an average plasma cotinine concentration of 119 ± 75 ng/mL^20^. Thus, cotinine, a specific nicotine metabolite that has an extended half-life in body fluids, is a direct biomarker of SHS exposure^21,22^.

In our study, the concentration of nicotine metabolites in breast milk reached 0.22 ng/mL among the women exposed to P-SHS. Nicotine metabolite concentrations in breast milk were correlated directly with SHS exposure; levels often registered above the limit of detection but below the cutoff established for active smokers^17^. Although a cutoff of 4.47 ng/mL (according to adult female serum data) is sometimes used to differentiate people who actively smoke from those with only SHS exposure, its utility is complicated by metabolic differences, exposure amount, and sample matrix variation^23,24^. Our findings definitively confirm that nicotine metabolites are detectable in the breast milk of non-smoking mothers exposed to SHS, although the resulting concentrations are potentially low. Future research should include a longitudinal follow-up study of the infant cohort to determine whether the observed early deficits in birth weight and length persist or whether compensatory catch-up growth occurs. This information can clarify the long-term developmental effects of prenatal and postnatal nicotine exposure. Furthermore, the biological mechanisms underlying shorter infant length should be investigated, specifically with regard to potential effects on bone growth or growth hormone pathways.

### Nicotine metabolite levels in maternal urine and breast milk in association with infant birth weight and length

In accordance with previous studies^9^, our findings indicate a strong, significant, and positive association between nicotine metabolite levels in maternal urine and breast milk; for every 1-unit increase observed in urine, breast milk concentration is predicted to rise by 2.071 units. Our findings specifically confirm the transfer of nicotine and its primary metabolite, cotinine, from maternal blood to breast milk; thus, these measurements in both matrices can be used for the direct assessment of infant exposure via breastfeeding^9^. Moreover, nicotine is transferred to breastfed infants from mothers who smoke or use snuff; the mean intake is 7 μg/kg/day via breast milk, and nicotine concentrations are inversely related to the interval between maternal nicotine intake and breastfeeding^25^. Thus, nicotine exposure during pregnancy might be strongly and significantly reflected in breast milk (β=2.071).

Our data support previous findings, suggesting that breast milk is the dominant route of SHS exposure in infants and that its contribution to SHS exposure exceeds that of environmental exposure alone^9^.

Infant weight was significantly and positively associated with nicotine metabolite levels in maternal urine. However, the regression coefficient (b) appears as 0.00 due to the extremely small scale of the measurement unit (e.g. 1 ng/mL). This negligible effect size suggests that although the association is statistically real (i.e. not due to chance), it lacks clinical or practical relevance. Therefore, within the context of this study, nicotine exposure during pregnancy did not significantly influence infant weight. Despite this finding, compelling evidence from other studies indicates that prenatal smoking exposure leads to lower birth weight and an increased risk of obesity (via ‘catch-up’ growth) later in infancy (at ages 6–14 months)^26^. Several previous studies have established a significant association between maternal urine nicotine metabolites, especially cotinine, and reduced infant birth weight^3,4,27^. Furthermore, the inconsistencies between our findings and previous studies may be attributed to confounding variables and differences in sample size.

The finding of a statistically significant negative association between nicotine metabolite levels in maternal urine and infant length is noteworthy: specifically, the data indicate that a 1-unit increase in urinary nicotine metabolites during pregnancy decreases infant length by 0.208 units. Unlike the clinically negligible finding for infant weight, this result suggests that higher nicotine exposure during gestation may be genuinely linked to measurable growth impairment in infants. In a previous study, active maternal smoking was associated with a significant reduction in birth length^28^: specifically, increased urinary cotinine concentration during pregnancy was associated with reduced infant birth length, which was quantified as a 0.25 ± 0.05 cm decrease for every 1000 ng increase in mean nicotine concentration^29^. In the Asian longitudinal cohort, high prenatal cotinine levels were associated with persistently shorter stature in offspring from birth through early childhood^30^. Moreover, maternal active and passive smoking during pregnancy, along with postnatal secondhand smoke exposure, independently increase the risk of wheezing in children up to two years of age^31^.

Our study hypothesis – that nicotine level in maternal urine serves as a more reliable predictor of nicotine metabolite transfer into breast milk than does self-reporting – was proved correct, and accounting for P-SHS-related variables enhanced the prediction of infant birth weight. By quantifying the minimal effect on weight but highlighting the concern regarding length, our findings enable clinicians and mothers to make informed decisions about prenatal and perinatal care.

### Influence of P-SHS-related variables on maternal nicotine metabolite levels

Nicotine metabolite levels in maternal urine are associated with breast milk nicotine levels, infant birth weight, and infant length. To understand the determinants of this exposure risk, this study assessed P-SHS-related variables, finding that indicators of greater SHS exposure were significantly linked to higher nicotine metabolite levels in both maternal urine and breast milk. As in previous studies, greater frequency (5–7 days/week) and intensity (>11 cigarettes/day) of family smoking significantly increase nicotine exposure in infants and children, thereby escalating their health risks^32^. In addition, in non-smoking women exposed to heavy SHS, maternal nicotine metabolite levels are influenced by several variables, including genetics (nicotine metabolism rate), race/ethnicity, age, and household/environmental factors^33^. Moreover, results of one study indicated that unmarried mothers were more likely to have higher cotinine levels than were married mothers^34^. A complete smoking ban in the home is the only measure that prevents SHS exposure in non-smoking residents. Unlike partial bans, comprehensive prohibition eliminates exposure from all indoor sources (air, surfaces, and clothing), thereby maximizing protection^35^. Because exposure levels are directly correlated with the presence and number of household smokers, a 100% ban on indoor smoking must be established and enforced^36^.

The results confirm a clear link between external SHS exposure and levels of nicotine in breast milk. The strongest predictors are the frequency of exposure (5–7 days/week) and prolonged exposure in the household locations (living room, bedroom, or both) where smoking occurs. The extremely large values (e.g. 6.577) correspond to exceptionally high odds ratios (725, derived from e^6.577^), meaning that the odds of having high levels of nicotine in breast milk are hundreds of times greater for mothers exposed to SHS 5–7 days a week than for those who are not so exposed; thus, the risk of SHS transfer is high. Furthermore, nicotine levels in breast milk are significantly affected by maternal active smoking status, the time elapsed between smoking and breastfeeding, and the intensity of SHS exposure^37^.

The research question is whether nicotine metabolite levels associated with urine and breast milk and P-SHS exposure variables have been identified as key risk factors in household locations within low-income urban communities. In addition, according to some studies, more than half of the variation in infant urinary nicotine levels could not be predicted by current statistical models; therefore, the contribution of persistent thirdhand smoke, potential dermal and oral routes of nicotine exposure, and shifts in public perception of smoking risk, should be explored^17^.

### Limitations

This study has several limitations. The cross-sectional design of this study prevents establishing causality between risk factors and outcomes, including the potential inverse association between breast milk nicotine metabolite exposure duration and infant birth weight and length. Additionally, the reliance on self-reported P-SHS variables may have introduced underestimation and recall bias. Furthermore, conducting this study at a single site in a low-income urban community in Bangkok, Thailand, may limit the generalizability of our findings to other regions. The study sample was drawn from a specific subpopulation, limiting its representativeness of the general population due to Berkson’s bias. Consequently, this selection bias can lead to misleading conclusions regarding the existence or strength of associations between variables. Despite these limitations, this study investigates the impact of breast milk nicotine metabolite levels on infant birth weight and length.

## CONCLUSIONS

The findings revealed that among primigravid women in the third trimester who lived in a low-income urban community in Bangkok and who were exposed to household smoking, greater SHS exposure significantly increased nicotine metabolite levels in both prenatal urine and postnatal breast milk, which in turn affected infant birth length. Furthermore, while infant weight was positively associated with maternal urinary nicotine metabolites, the near-zero regression coefficient indicates a lack of clinical relevance. P-SHS-related variables reflecting greater SHS exposure (e.g. family smoking frequency of 5–7 days a week, cohabitation with people who smoked heavily, and smoking in the household) were significantly associated with higher nicotine metabolite levels in prenatal urine and postnatal breast milk, thereby increasing the exposure risk for the mother and infant.

## Supplementary Material



## Data Availability

The data supporting this research are available from the authors on reasonable request.

## References

[1] Pineles BL, Park E, Samet JM. Systematic review and meta-analysis of miscarriage and maternal exposure to tobacco smoke during pregnancy. Am J Epidemiol. 2014;179(7):807-823. doi:10.1093/aje/kwt334 PMC3969532 24518810

[2] Suzuki D, Wariki WMV, Suto M, et al. Secondhand smoke exposure during pregnancy and mothers’ subsequent breastfeeding outcomes: a systematic review and meta-analysis. Sci Rep. 2019;9(1):8535. doi:10.1038/s41598-019-44786-z PMC6562041 31189894

[3] Vojisavljevic D, Rudd D, Smith R, Kandasamy Y. The relationship between maternal smoking and infant birth weight: improving accuracy through urine cotinine analysis and effective medical record strategies. Children (Basel). 2024;11(8):1028. doi:10.3390/children11081028 PMC11353045 39201962

[4] Schechter J, Do EK, Zhang JJ, et al. Effect of prenatal smoke exposure on birth weight: the moderating role of maternal depressive symptoms. Nicotine Tob Res. 2020;22(1):40-47. doi:10.1093/ntr/nty267 PMC7297019 30590728

[5] Hamadneh S, Hamadneh J. Active and passive maternal smoking during pregnancy and birth outcomes: a study from a developing country. Ann Glob Health. 2021;87(1):122. doi:10.5334/aogh.3384 PMC8641528 34900622

[6] Kummet CM, Moreno LM, Wilcox AJ, et al. Passive smoke exposure as a risk factor for oral clefts-a large international population-based study. Am J Epidemiol. 2016;183(9):834-841. doi:10.1093/aje/kwv279 PMC4851990 27045073

[7] Leng S, Yang D, Li W, Liu Z, Li H. The longitudinal association between second-hand smoke exposure and maternal depression among non-smoking pregnant women in East China: a prospective birth cohort study. Public Health. 2025;244:105760. doi:10.1016/j.puhe.2025.105760 40378719

[8] Goldade K, Nichter M, Nichter M, Adrian S, Tesler L, Muramoto M. Breastfeeding and smoking among low-income women: results of a longitudinal qualitative study. Birth. 2008;35(3):230-240. doi:10.1111/j.1523-536X.2008.00244.x PMC2830716 18844649

[9] Primo CC, Ruela PB, Brotto LD, Garcia TR, Lima Ede F. Effects of maternal nicotine on breastfeeding infants. Rev Paul Pediatr. 2013;31(3):392-397. doi:10.1590/S0103-05822013000300018 PMC4182966 24142324

[10] Kunno J, Luangwilai T, Manomaipiboon B, et al. The urban health themes and urban factors associated with health: a brief review: status quo in urban health and its factors associated with health. Vajira Med J. 2024;68(4):e269743. doi:10.62691/vmj.2024.269743

[11] Kunno J, Luangwilai T, Pimviriyakul P, et al. Active smoking in urban households: an association between urinary cotinine metabolite level and serum eGFR concentration. Tob Induc Dis. 2024;22(April):59. doi:10.18332/tid/186071 PMC10996036 38586496

[12] Kunno J, Pimviriyakul P, Luangwilai T, et al. Effect of children secondhand smoke exposure associated with GABA concentration: influence from parents who are extremely heavy smokers in urban households. Sci Total Environ. 2024;918:170720. doi:10.1016/j.scitotenv.2024.170720 38325467

[13] Reece S, Morgan C, Parascandola M, Siddiqi K. Secondhand smoke exposure during pregnancy: a cross-sectional analysis of data from demographic and health survey from 30 low-income and middle-income countries. Tob Control. 2019;28(4):420-426. doi:10.1136/tobaccocontrol-2018-05428 PMC10442074 30026189

[14] Espy KA, Fang H, Johnson C, Stopp C, Wiebe SA. Prenatal tobacco exposure: developmental outcomes in the neonatal period. Dev Psychol. 2011;47(1):153-156. doi:10.1037/a0020724 PMC3057676 21038943

[15] Karvonen M, Saari A, Sund R, Sankilampi U. Maternal smoking during pregnancy and offspring head growth in comparison to height and weight growth up to 6 years of age: a longitudinal study. Clin Epidemiol. 2021;13:959-970. doi:10.2147/CLEP.S327766 PMC8520481 34675684

[16] Rasmussen KM, Yaktine AL, Institute of Medicine (US) and National Research Council (US) Committee to Reexamine IOM Pregnancy Weight Guidelines, eds. Weight Gain During Pregnancy: Reexamining the Guidelines. National Academies Press (US); 2009. 20669500

[17] Parks J, McLean KE, McCandless L, et al. Assessing secondhand and thirdhand tobacco smoke exposure in Canadian infants using questionnaires, biomarkers, and machine learning. J Expo Sci Environ Epidemiol. 2022;32(1):112-123. doi:10.1038/s41370-021-00350-4 PMC8770125 34175887

[18] Sobh E, Mohammed AM, Adawy Z, Nassef AH, Hasheesh A. The impact of secondhand smoke exposure on the pregnancy outcome: a prospective cohort study among Egyptian community. Egypt J Bronchol. 2021;15(1):50. doi:10.1186/s43168-021-00097-4

[19] Loukopoulou AN, Vardavas CI, Farmakides G, et al. Counselling for smoking cessation during pregnancy reduces tobacco-specific nitrosamine (NNAL) concentrations: a randomized controlled trial. Eur J Midwifery. 2018;2(November):14. doi:10.18332/ejm/99546 PMC7846038 33537575

[20] Dempsey D, Jacob P 3rd, Benowitz NL. Accelerated metabolism of nicotine and cotinine in pregnant smokers. J Pharmacol Exp Ther. 2002;301(2):594-598. doi:10.1124/jpet.301.2.594 11961061

[21] Ramdzan AN, Almeida MIGS, McCullough MJ, Segundo MA, Kolev SD. Determination of salivary cotinine as tobacco smoking biomarker. TrAC Trends Anal Chem. 2018;105:89-97. doi:10.1016/j.trac.2018.04.015

[22] He J, Wu Z, Liang Y, He J. Exposure to secondhand smoke and physical disabilities in non-smokers: a national cross-sectional study with cotinine measurements from NHANES 2013-2018. Tob Induc Dis. 2025;23(February):18. doi:10.18332/tid/200546 PMC11843551 39989509

[23] Geraghty SR, McNamara K, Kwiek JJ, et al. Tobacco metabolites and caffeine in human milk purchased via the internet. Breastfeed Med. 2015;10(9):419-424. doi:10.1089/bfm.2015.0096 PMC4638204 26394021

[24] Lee HS, Cho JH, Lee YJ, Park DS. Effect of second-hand smoke exposure on establishing urinary cotinine-based optimal cut-off values for smoking status classification in Korean adults. Int J Environ Res Public Health. 2022;19(13):7971. doi:10.3390/ijerph19137971 PMC9265992 35805637

[25] Dahlstrom A, Ebersjo C, Lundell B. Nicotine exposure in breastfed infants. Acta Paediatr. 2004;93(6):810-816. doi:10.1111/j.1651-2227.2004.tb03023.x 15244232

[26] Sowan NA, Stember ML. Effect of maternal prenatal smoking on infant growth and development of obesity. J Perinat Educ. 2000;9(3):22-29. doi:10.1624/105812400X87734 PMC1595035 17273214

[27] Reeves S, Bernstein I. Effects of maternal tobacco-smoke exposure on fetal growth and neonatal size. Expert Rev Obstet Gynecol. 2008;3(6):719-730. doi:10.1586/17474108.3.6.719 PMC2770192 19881889

[28] Silva AI, Camelo A, Madureira J, et al. Urinary cotinine assessment of maternal smoking and environmental tobacco smoke exposure status and its associations with perinatal outcomes: a cross-sectional birth study. Environ Res. 2022;203:111827. doi:10.1016/j.envres.2021.111827 34363802

[29] Wang X, Tager IB, Van Vunakis H, Speizer FE, Hanrahan JP. Maternal smoking during pregnancy, urine cotinine concentrations, and birth outcomes. A prospective cohort study. Int J Epidemiol. 1997;26(5):978-988. doi:10.1093/ije/26.5.978 9363518

[30] Ng S, Aris IM, Tint MT, et al. High maternal circulating cotinine during pregnancy is associated with persistently shorter stature from birth to five years in an Asian cohort. Nicotine Tob Res. 2019;21(8):1103-1112. doi:10.1093/ntr/nty148 PMC6211655 30032178

[31] Vardavas CI, Hohmann C, Patelarou E, et al. The independent role of prenatal and postnatal exposure to active and passive smoking on the development of early wheeze in children. Eur Respir J. 2016;48(1):115-124. doi:10.1183/13993003.01016-2015 26965294

[32] Joseph DV, Jackson JA, Westaway J, Taub NA, Petersen SA, Wailoo MP. Effect of parental smoking on cotinine levels in newborns. Arch Dis Child Fetal Neonatal Ed. 2007;92(6):F484-F488. doi:10.1136/adc.2006.108506 PMC2675401 17580319

[33] Hawkins SS, Dacey C, Gennaro S, et al. Secondhand smoke exposure among nonsmoking pregnant women in New York City. Nicotine Tob Res. 2014;16(8):1079-1084. doi:10.1093/ntr/ntu034 PMC4110923 24642590

[34] Wheeler DC, Boyle J, Barsell DJ, et al. Neighborhood deprivation is associated with increased risk of prenatal smoke exposure. Prev Sci. 2022;23(7):1078-1089. doi:10.1007/s11121-022-01355-7 PMC9385886 35179695

[35] Tripathi O, Parada H Jr, Shi Y, et al. Perception of harm is strongly associated with complete ban on in-home cannabis smoking: a cross-sectional study. BMC Public Health. 2024;24(1):669. doi:10.1186/s12889-024-18072-1 PMC10908115 38429696

[36] Cheng KW, Glantz SA, Lightwood JM. Association between smokefree laws and voluntary smokefree-home rules. Am J Prev Med. 2011;41(6):566-572. doi:10.1016/j.amepre.2011.08.014 PMC3222862 22099232

[37] Vlachou M, Kyrkou GA, Vivilaki V, et al. Tobacco smoke exposure and lactation. Cureus. 2024;16(11):e73651. doi:10.7759/cureus.73651 PMC11645517 39677116

