# Supplementary material for: Secondhand smoke exposure during pregnancy: Nicotine metabolites in maternal urine and breast milk as biomarkers of infant birth weight and length, a cross-sectional study
Source: Tob Induc Dis. 2026 Sep 17;24:10.18332/tid/226408. doi: 10.18332/tid/226408 (PMC13584920; doi:10.18332/tid/226408)

Tobacco Induced Diseases

Supplementary file

© 2026 Kunno J. et al.

DOI:
10.18332/tid/226408

The content has been provided by the author(s) and has not been reviewed, verified, or endorsed by European Publishing. It may not have undergone peer review. The views, opinions, and recommendations expressed are solely those of the author(s) and do not necessarily reflect the position of European Publishing. European Publishing accepts no responsibility or liability for any consequences arising from the use of, or reliance on, this content.

Supplemental File 1. Conceptual framework of the P-SHS exposure pathway

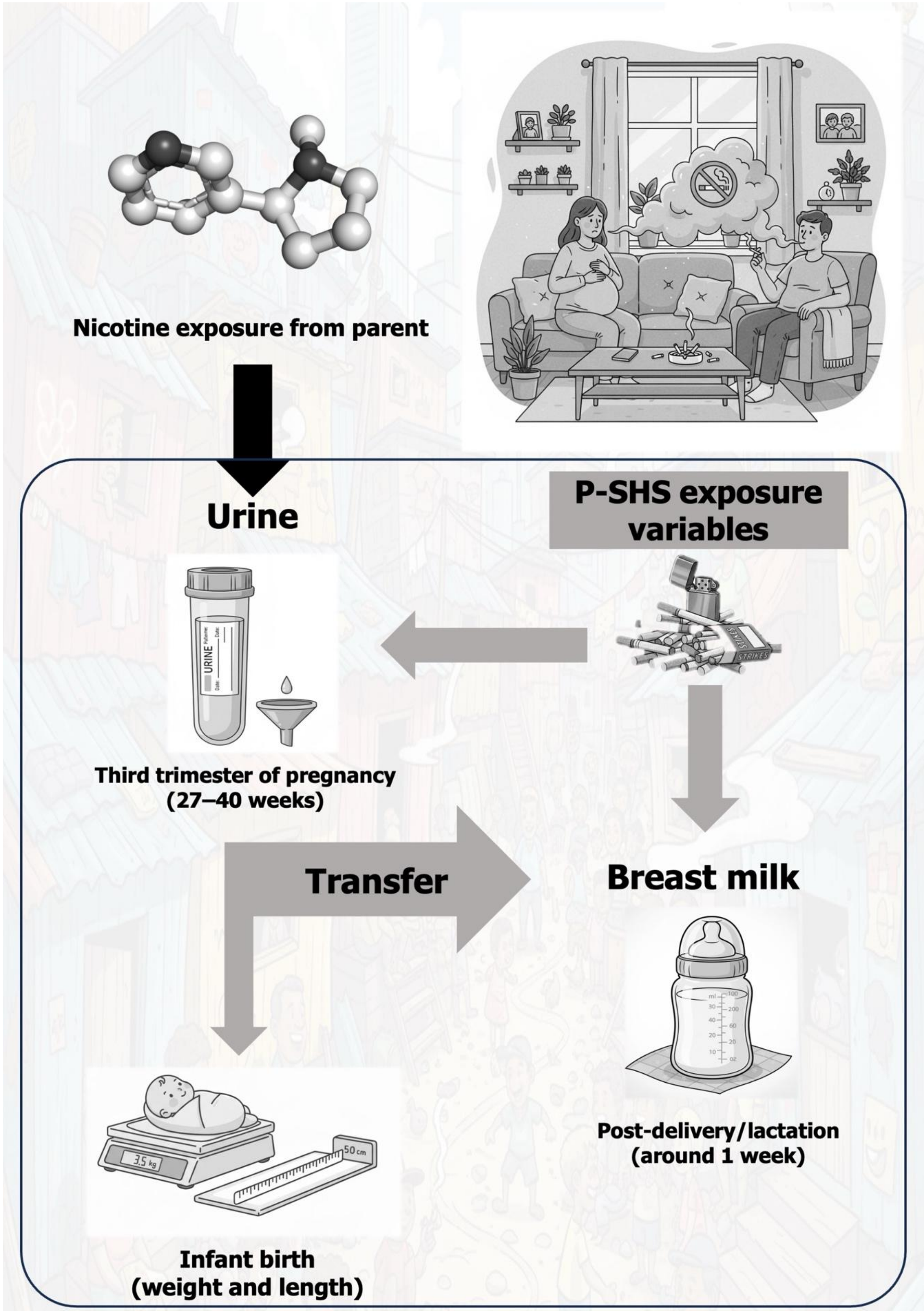

Supplemental File 2. Flowchart of the data collection and participant classification

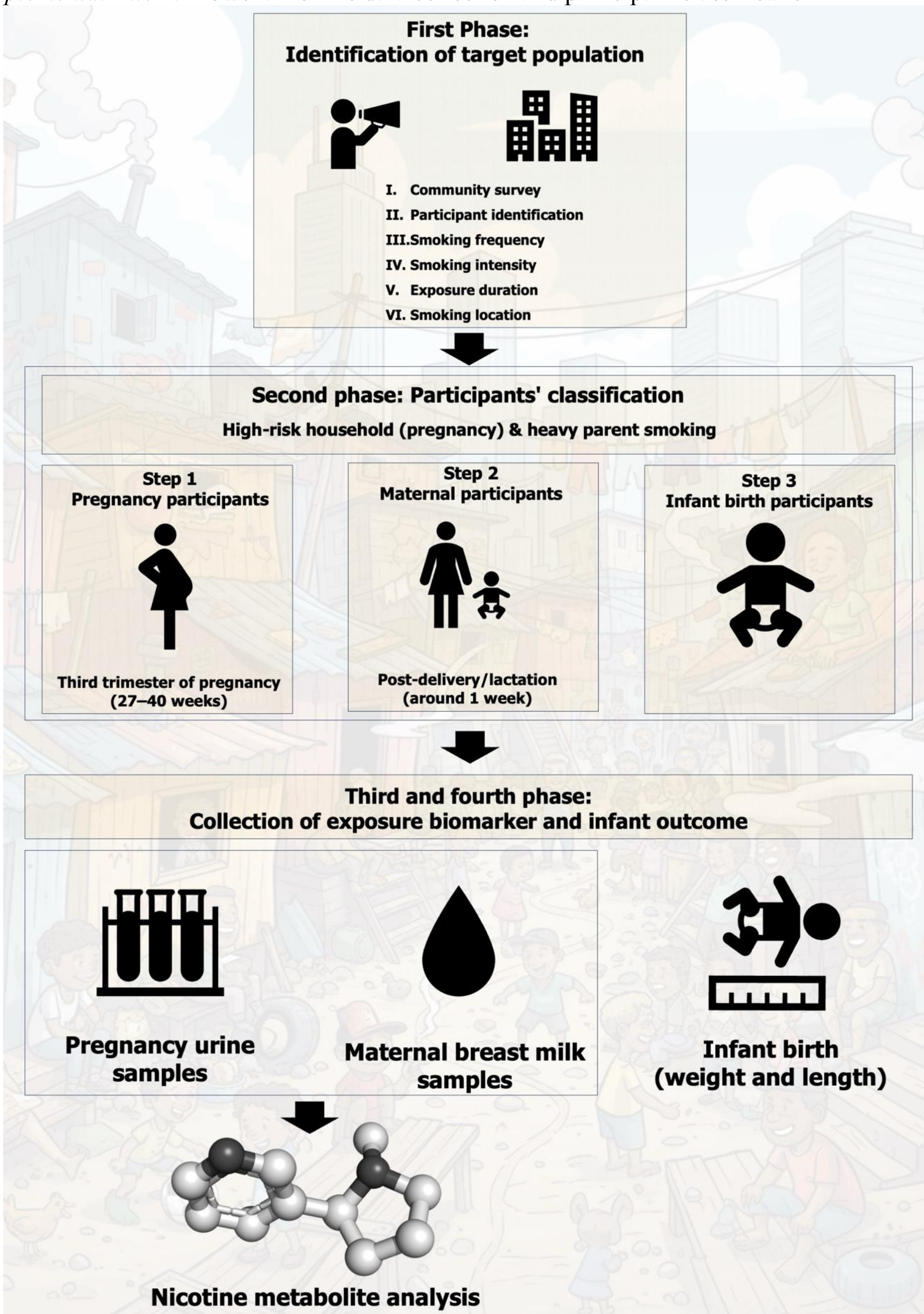

Supplement: Supplementary file 1 [file TID-24-166-s1.pdf]
